# The Thrill of Speedy Descents: A Pilot Study on Differences in Facially Expressed Online Emotions and Retrospective Measures of Emotions During a Downhill Mountain-Bike Descent

**DOI:** 10.3389/fpsyg.2019.00566

**Published:** 2019-04-02

**Authors:** Audun Hetland, Eirik Kjelstrup, Matthias Mittner, Joar Vittersø

**Affiliations:** Department of Psychology, UiT The Arctic University of Norway, Tromsø, Norway

**Keywords:** emotion, facial expression, online emotions, functional wellbeing approach, extreme sport, downhill mountain biking

## Abstract

When extreme sport athletes explain the engagement behind their taxing and risky endeavors, they often refer to the happiness generated by the activities. However, during the activity, these athletes seem neither pleased nor happy. This article proposes some answers from a study of facially expressed emotions measured moment by moment during downhill mountain biking. Self-reported emotions were also assessed immediately after the trip was over. The participants display less happiness during the activity than before and after the activity. No significant associations between facially expressed and self-reported emotions were observed. Findings are discussed with reference to the functional well-being approach arguing that some momentary feelings are non-evaluative in the sense of being caused by the difficulty of the ongoing activity. Within this framework, easy tasks produce happy feelings while difficult tasks produce interest—regardless of whether a goal has been reached or not. By contrast, retrospective emotions involve the evaluation of the activity in relation to its goal. When a goal is accomplished, the accompanying feeling is positive. If a goal (or value) is threatened, lost, or not achieved, negative feelings follow.

## Introduction

When BASE jumper Ryan Saunders landed successfully after a particularly challenging jump, he celebrated the achievement with a verbal eruption that lasted over 60 s: “Yes! Yes! F…ing yes! I don’t care if I sound like the biggest retard in the world – that was f…ing awesome!” (NRK, 2011). Observing the scene leaves no room for doubt about Ryan’s emotional state—he was as happy as a human being can be. Indeed, studies show that the feeling of happiness is among the key motivators of extreme sport (Brymer, 2005; Willig, 2008; Brown and Fraser, 2009; Hetland and Vittersø, 2012). See Kerr and Houge Mackenzie (2012) or Woodman et al. (2010) for an extended list of motives.

A bit of reflection, however, should bring forward the question of why such taxing and dangerous endeavors make people happy. Flow theory (e.g., Csikszentmihalyi, 1999) argues that these positive feelings only appear after the activity has ended (Moneta and Csikszentmihalyi, 1996). By contrast, the functional wellbeing approach (FWA; Vittersø, 2013, 2016, 2018) argues that positive feelings are actually present during flow, but that they are not experienced as pleasure or happiness but rather emotions like interest, immersion, and engagement. Following this theory, the feeling of happiness comes later, as the result of an appraisal of goal accomplishment.

In a previous study, we have used small, head-mounted video cameras to capture backcountry skiers facially displayed emotions, while they were skiing (Hetland et al., 2018 – this issue). The results showed that the participants displayed significantly less happiness while skiing than when they stopped to take a break. These results offer some support to the assumption that happiness is not generated on a moment-by-moment basis but arises after the activity has ended. Further, following the functional well-being approach, the feelings that people actually experience during such concentrated moments of challenging activities are perhaps better described as interest and engagement. Here, we refer to “interest” as an emotion, typically described as “a feeling of wanting to investigate” (Izard and Ackerman, 2000), or “engaged curiosity” (Panksepp, 1998, p. 149). Functionally, it is assumed that interest is experienced in order to foster the development of competence and skills (e.g. Tomkins, 1962; Fredrickson, 1998; Silvia, 2006; Thoman et al., 2011; Smith et al., 2014; Renninger and Hidi, 2015).

Interest is not included as one of Ekman’s basic emotions (Ekman, 1993), which is the basis for the previously used facial analysis software and thus not natively included. Therefore, we were previously unable to test if the participants did, in fact, experience interest during the activity. To compensate for this limitation, we implemented here a measure of facially displayed interest, based on the work of Campos et al. (2012), who proposed a method to measure facially expressed interest based on a new combination of action units that describes facial movements.

Our previous study of backcountry skiers applied an entirely new way of capturing emotions during an extreme sport activity. We apply the same method to a new population, this time on a sample of downhill mountain bikers. In the present article, we seek to (1) replicate our main finding that happiness arises after the activity is over and (2) try to capture displayed interest to possibly test the assumption that difficult activities are characterized of momentary interest and retrospective happiness. We test this new measure on a sample of downhill mountain bikers while they bike.

### Extreme Sport

Extreme sport is not easy to define, and the term is often used interchangeably with other terms such as action sport, adventure sport, and lifestyle sport (Puchan, 2004; Brymer and Schweitzer, 2013). The number of people participating in different extreme sports is growing all over the western world (Thorpe and Wheaton, 2013). According to Willig (2008), extreme sport can be defined as recreational physical activities that carry a risk of serious physical injury or even death. This definition includes activities such as BASE jumping, skydiving, hang gliding and paragliding, mountain climbing, surfing, white water kayaking, backcountry skiing, and mountain biking.

As one of many extreme sports, downhill mountain biking has increased significantly in popularity the past two decades. The sport is based on descending steep, rough terrain at high speed using heavy, specialist bikes with long-travel suspension. A downhill mountain biker needs to master technical skills to handle obstacles such as elevated, narrow wooden board ways, log rides, ladder bridges, and different drops and jumps. Falling of the bike in high speed may cause severe injury to the participants (Becker and Moroder, 2017).

Such voluntary exposure to extreme hazards has received increased scientific attention, and the list of potential motives is increasing (Celsi et al., 1993; Willig, 2008; Brymer and Gray, 2009; Woodman et al., 2010; Hetland and Vittersø, 2012; Kerr and Houge Mackenzie, 2012; Barlow et al., 2013; Arijs et al., 2017; Frühauf et al., 2017; Holmbom et al., 2017; Hetland et al., 2018). Among the important motives is the experience of positive emotions often explained in the context of flow (Delle Faveaaa et al., 2003).

### Positive Emotions

A flow state is most likely to occur when the individual is intrinsically motivated to perform a given task (Csikszentmihalyi, 1975). Such motivational states are sometimes referred to as autotelic, meaning that the activity has no external goal beyond performing the activity in itself. Several criteria are considered important for a flow state to occur. Three of these are of special importance: (1) clear goals to the activity, (2) constant feedback on your performance, and (3) a balance between the challenge and the experienced skills.

However, some researchers argue that a balance between challenge and skills may not be the ideal conditions for a flow state to occur. Løvoll and Vittersø (2014), for example, argue that a small imbalance between challenges and skills creates a more optimal situation for flow. An imbalance, they argue, will lead the actor to experience emotions like interest and not pleasure and also lead the participants to be more focused and to perform their best in the given situation – which in turn may create better conditions for skill development.

Several scholars therefore point out the usefulness of separating feelings of pleasure from feelings of interest (Hidi and Renninger, 2006; Vittersø et al., 2010; Renninger and Hidi, 2011; Vittersø, 2016). Pleasure, the argument goes, works as a reward when reaching small or big goals, while interest facilitates learning, growth, and the struggle toward reaching difficult goals. In the functional wellbeing approach (FWA; Vittersø, 2016), these systems are supposed to fulfill two important needs: the need for stability (pleasure) and the need for change (interest).

The rich literature on homeostatic regulation has established as a fact that feelings of pleasure and satisfaction are internal messages about a homeostatic stability that has been reestablished. Examples of such reestablished stabilities are needs being fulfilled or goal being accomplished (Denton, 2005; Damasio, 2018). Given the high correlations often observed between pleasure and happiness (Russell, 1980; Vittersø, 2016), it seems reasonable to assume that the emotion of happiness fulfills a function somewhat similar to that of pleasure and satisfaction. For example, a study by Shaver et al. (1987) showed that the most common cause of a happy emotion reported by their participants was the accomplishment of a desired outcome. Similarly, studies since Darwin (1872) have recognized how happiness broadcasts to us a signal about one’s environment, and social circumstances in particular, being benign and favorable. Mentally then, happiness is an emotion about wellbeing. The message from a happy mind is that the current state of being is a good one; no urgent changes are required (Oatley, 1992; Wierzbicka, 1999; Ortony et al., 2005; Keltner, 2009).

By contrast, feelings such as interest and engagement keep the organism alerted and ready for effortful action. These feelings motivate activities to continue over time and during hardship, thus enabling the organism to postpone the return to homeostatic equilibrium. For example, Thagard (2002) carefully noted what kind of emotions James Watson reported in his book on the discovery of the DNA (Watson, 1999), when Watson and Crick struggled to understand the structure of the molecule. Thagard observed that feelings such as interest, curiosity, and wonder were common during phases of investigation and the generation of new research questions. Feelings such as pleasure, happiness, and beauty, on the other hand, were typically reported in phases of discovery and when important hypotheses were justified.

This view also has support in the field of neuroscience. A study by Burgdorf and Panksepp (2006) showed that endogenous opioids are involved in the regulation of the hedonic emotions such as pleasure, satisfaction, and happiness, whereas dopamine is important for eudaimonic emotions such as interest, engagement, and enthusiasm. The FWA also draws a distinction between momentary emotional experiences and the overall evaluation of an event (Vittersø, 2016). Momentary emotions are created by the execution of small acts toward a goal. Overall emotions, however, arise as a response to goal accomplishment for the activity as a whole. If a goal has been fully or partly achieved, the subject will be rewarded with a pleasant feeling of satisfaction, happiness, or mastery. However, if the goal is not achieved, the evaluation of the experience will result in displeasure and negative feelings.

### Measuring Emotions

There are several ways in which emotions can be understood, and thus, how they can be measured. Several researchers separate between two different levels of emotional experience (see, for example, Nilsen and Kaszniak, 2007; Vittersø, 2013, 2018). The first level concerns the emotions experienced from moment to moment, which might sometimes be non-evaluative. The second level is retrospective, and these emotions always include a cognitive evaluation. In the current article, these emotions will be referred to as online and retrospective emotions.

The momentary and retrospective levels of emotional experience may often correlate, but they are also likely to differ. The retrospective emotions might often be biased by situational or social aspect of the situation, which have been shown to alter the actual online perspective itself (Schooler et al., 2003). In addition, high arousal, inherent in extreme sport has been reported to impair immediate emotional reports (Corson and Verrier, 2007). Time, on the other hand, has also been shown to affect retrospective emotional report (Robinson and Clore, 2002; Clore and Robinson, 2012).

Given these challenges, are there ways to measure emotions online without them being affected of the process of measuring? Kahneman and Riis (2005) have suggested that use of electroencephalography (EEG), heart rate (HR), and skin conductance might give good measures of emotional experience. Other studies have also used fMRI scanners to prove that different regions of the brain are associated with different emotional reactions. Although providing a good measure of online emotional experience, these methods are in most cases limited to a laboratory setting.

Another way of measuring online emotions is through capturing and analyzing facial expressions. In a similar study to the one described in this article, Hetland et al. (2018) filmed and analyzed the facial expressed emotions of a sample of backcountry skiers while they descended a mountain on skis. The participants’ facially expressed emotions were analyzed using automatic facial coding (AFC) software. This method provided a moment-by-moment measure of emotions, which did not need a cognitive interaction with any kind of apparatus.

### Facially Expressed Emotions

According to Ekman (1993), there are at least six basic emotions (happiness, fear, anger, surprise, disgust, and sadness), and these are expressed with distinctly different facial expressions. These expressions are (1) innate (Tracy and Matsumoto, 2008), (2) universal, (3) reliable measures of the subjective experience of the individual, and (4) correlate to the physiological and expressive components related to the given emotion (Matsumoto et al., 2008).

In order to get reliable measures of facial expressions, Ekman and Friesen (1978) developed the facial action coding system (FACS), a system that is based on measuring activity and movement in different facial muscles. FACS is based on 46 different action units (AUs), each of which is responsible for a particular facial movement. As an example, upward movement of the lip corners would characterize a smile and the movement is being controlled by the muscle zygomaticus major (Ekman and Friesen, 1978). In FACS, this would be described as activation in AU6 (cheek raiser) and AU12 (lip corner puller).

In a study by Campos et al. (2012), the authors provide a description of seven different positive facially expressed emotions, including facial expressed interest. A closer look shows that the facial expression in which Campos and his colleagues interpret as representing interest shares distinct qualities with the facial expression of sadness (Ekman, 1993). In Hetland et al. (2018) study, sadness was the second most prominent online emotion after happiness. However, the participant’s self-reported emotions showed low levels of sadness. In a previous study on BASE jumpers and skydivers (Hetland and Vittersø, 2012), none of the participants reported any sadness but high levels of interest throughout the jump.

The similarities between displayed sadness and interest are striking. Both interest and sadness are characterized by activation in AU1 (inner brow raise) and AU4 (brow lowered). The only difference is found in mouth gestures, where interest is described by activation of AU24 (lip pressured), where sadness is expressed by activation of AU15 (lip corn puller).

### Automatic Facial Coding

In the past, facial expressions were usually coded manually by trained raters. However, as technology has improved, this can now be done automatically. Automatic facial coding (AFC) has several advantages and a few disadvantages, compared to manual coding. First and foremost, automatic capturing, interpreting, and coding demand very little labor. In addition, different studies have shown that such digital analyses outperform non-expert coders and are approximately as accurate as expert coders (Bartlett et al., 1999; Terzis et al., 2010; Lewinski et al., 2014).

In a reliability study, Lewinski et al. (2014) found that FaceReader, the software used in the present study, recognized 88% of the targeted emotions in both the Warsaw Set of Emotional Facial Expression (WSEFEP; Olszanowski et al., 2014) and the Amsterdam Dynamic Facial Expression Set (ADFES; van der Schalk et al., 2011). The corresponding number for human raters was 85%.

On the downside, the methods for coding facial expression are still developing, particularly in how they distinguish different positive emotions. It takes time before enough pre-coded material is available for a potential update of the computer model used by AFC programs. In addition, humans are able to code other movements, body postures, partly occluded faces, and images with varying image quality or perspectives in which the software is not currently able to read.

Still, the advantages of automatic coding in most cases outweigh the cost, making this method increasingly popular. It has previously been used in a number of fields such as emotion science (Bartlett et al., 1999; Chentsova-Dutton and Tsai, 2010), educational research, human-computer interaction (Cohen et al., 2003), consumer behavior (Danner et al., 2014), user experience (Goldberg, 2014), clinical investigations of facial nerve grading in medicine (Dulguerov et al., 1999), monitoring pain (Lucey et al., 2012), reaction to advertisement and commercial films (Teixeira et al., 2012; Hetland et al., 2016), and emotions in extreme sport (Hetland et al., 2018).

### Aims of the Study

The overarching aims of the study are to (1) replicate the findings from Hetland et al. (2018) that happiness arises first after the activity is over, (2) test if the newly developed measure of online interest can provide us with a measure of online interest, and finally (3) to explore the relation between measures of online emotions during the activity and self-report emotions given immediately after the activity is over. Based on these aims, three hypotheses were put forward.

Hypothesis 1. Given the challenging nature of downhill mountain biking, and the rewarding role played by hedonic feelings when goals and sub-goals have been achieved, we refer to the FWA and predict that participants will experience more happiness immediately after they stop than when they are actively biking.

Hypothesis 2. On the same terms, the FWA predicts that participants will experience more interest during the activity than before and after.

Hypothesis 3. The FWA does not predict a strong relationship between the online emotions during a difficult event, and the self-reported emotions given when the activity is over.

## Materials and Methods

### Participants

Twenty-four participants (two females) were recruited through social media and snowball sampling. Age ranged from 19 to 46 (*M* = 27.13, *SD* = 6.81). The sample mostly not only consisted of Norwegian citizens (*n* = 21) but also included bikers from Sweden (*n* = 2) and Poland (*n* = 1). All participants gave written and informed consent before participating in the study.

### Procedure and Materials

The data for this study came from five different sources: two questionnaires assessing (1) background variables (questionnaire A) and (2) state emotions right after the activity (questionnaire B). These questionnaires were available in Norwegian and English and were translated and back translated to ensure equivalence across languages. Online emotions were measured with (3) automatic facial coding software. In addition, (4) heart-rate (HR) data and (5) speed were recorded during the activity. For this present study, only data from questionnaire B and the facially expressed emotions will be investigated.

Participants met up with the first author at various known DH locations, either by chance or after agreement. After signing the informed consent, participants were requested to fill out questionnaire A. When this was completed, all participants were given a special helmet, equipped with a front-mounted camera filming the face of the participant. They were also equipped with a heart-rate (HR) monitor that also recorded speed based on GPS coordinates. After synchronizing the HR monitor and the camera, participants were asked to perform a normal downhill run. Immediately after finishing their run, participants were given questionnaire B, which concluded the study. All questionnaires were answered on an Apple iPad, using the Qualtrics application.

### Facially Expressed Emotions

See Figure 2.

#### The Camera and Helmet Mount

**Figure 1:**
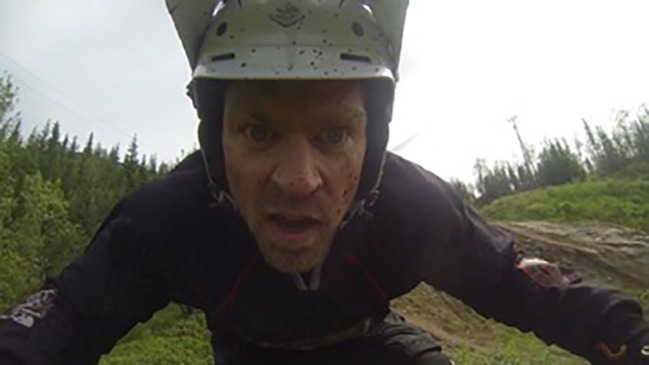
The view given by the face-fronting camera during a DH-descent. (The participant gave his consent to use this picture).

The participants were equipped with a GoPro 4 camera set to full HD (1,080) progressive, field of view (FOV) set to medium and a frame rate of 25 images per second. Based on these settings, the film consists of 25 self-contained still images per second where the entire face of the participants was visible (see Figure 1).

**Figure 2:**
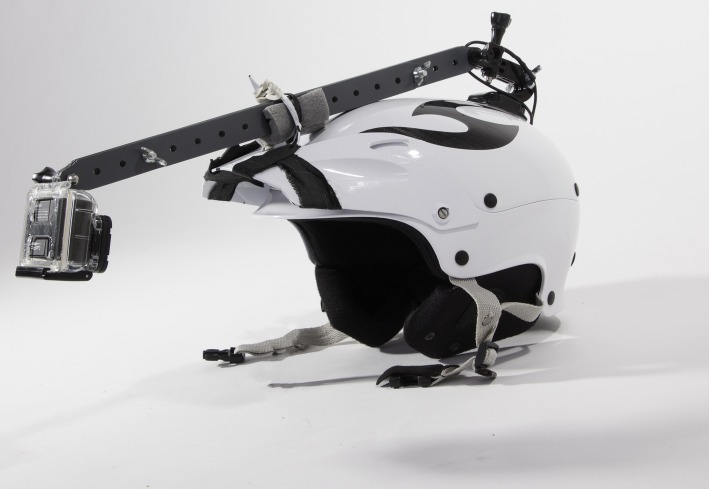
The helmet with camera setup.

The camera was mounted on the helmet with an extension rod connected to a standard helmet mount placed on the top of the helmet. The extension rod was also attached and supported sideways by an adapted helmet brim and fastened with rubber bands (see Figure 2). This setup has two advantages. First, it prevents the camera from hitting the participants’ face in case of a fall. Mounted as shown on the illustration, the camera can easily detach from the front support allowing it to swing up, or sideways but not downward. Second, the two points of attachment also make the camera mount very stable. In addition, we also had five different helmets in different sizes with additional padding if needed, to make sure that the helmet fit snugly to the participants’ head. This made sure that the camera would follow the head movement and thus effectively eliminating the potential problem of camera shake – which could else have been a potential problem for the analysis.

### The Technique

The videos from the camera capturing facial expressions were then analyzed using FaceReader version 6.0 (Noldus, 2015). This computer software bases its interpretations on the facial action coding system (FACS; Ekman and Friesen, 1978) when scoring facial expressions. To be able to automatically read facial expressions, the software creates and fits a three-dimensional model of the human face to the image. This model consists of a mesh with 491 different measure points that use an algorithmic approach to code movements based on the active appearance method (Cootes and Taylor, 2004). The model has been trained on approximately 10,000 images scored by FACS certified experts. The video was manually coded into different segments of (1) before the run, (2) during, and (3) after the run. The data were then exported into an excel file, before being analyzed further.

### The Variables

The automatic facial coding (AFC) software detects the face, applies, and adapts the mesh to individual features, and by measuring the ongoing muscle movement between the points automatically classifies the six basic emotional expressions of happiness, sadness, disgust, surprise, fear, and anger. In addition to the basic emotions, a neutral state is also recorded. All emotional scores are ranged from 0 (not present) to 1 (fully activated). The measured emotions are not mutually exclusive, meaning that more than one emotional expression can be present at the same time. The film was analyzed frame by frame, providing 175 measures every second, when taken all of the six emotions and the neutral state into account.

### Operationalizing a Measure of Facially Expressed Interest

A new feature in the FaceReader version 6.0 gave us the opportunity to export action units (AUs) in addition to the six facial expressed emotions as described previously. In FaceReader, these data are scored from A (slightly active) to E (fully activated). Non-active action units are scored as NaN. Based on the arguments given by Campos et al. (2012), we used AU1 (inner brow raiser) and AU4 (brow lowered) as a representation of facially expressed interest. To register as interest, the two action units had to be active simultaneously. The measure was given numeric scores from 0 (not active) to 1 (both AU scored at E, fully activated) in the following way: both AU activations as originally detected by FaceReader (A–E) where transformed to ranks (not activated = 0, …, *E* = 5), multiplied with each other and scaled to the [0,1] interval by division by the maximum possible score of 25. The item was scored 25 times each second, providing us with the same amount of measures as the original data.

### Self-Reported Emotions

Self-reported emotions were measured immediately after the activity. The participants reported five basic emotions. These five emotions were (1) happiness, (2) interest, (3) fear, (4) anger, and (5) sadness. To increase reliability of the scale, the two positive emotions, happiness, and interest were measured with three items each. For happiness, these were happiness, pleasure, and satisfaction. For interest, these adjectives were interest, engagement, and enthusiasm. The negative emotions were less important for our research goal and therefore measured with their single items: fear, anger, and sadness. The items were presented with the introduction “Now, let us look at your total experience of biking down the mountain. There are a number of emotions you may have experienced, to a varying extent. Try to recall how you felt while you were biking and check the number that best describes your emotions. Note that you have to answer the first nine items to be able to proceed.” For each of the items, the participants responded using an end-point labeled rating scale ranging from 1 (not at all) to 7 (very much).

## Results

### Duration of the Events

The measures of facially expressed emotions were divided into three different events of variable duration: before, during, and after the downhill ride. The mean recording duration before the downhill ride was *M* = 16.6 s (*SD* = 14.8 s), the mean duration for the downhill episode was *M* = 142 s (*SD* = 168 s), and the post-ride episode had a mean duration of *M* = 8.4 s (*SD* = 11.2).

### Facial Expressed Emotions Before, During, and After the Event

**Figure 3:**
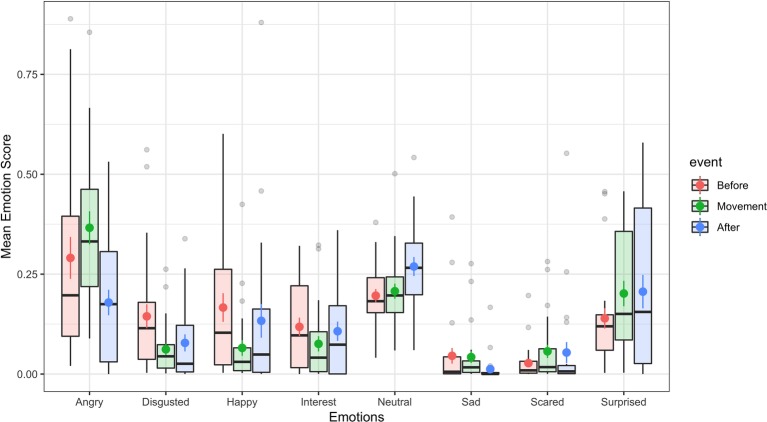
Boxplots of individual mean scores of emotions before, during (referred to as “movement”), and after the downhill ride. Box edges represent the first and third quartiles, and the solid middle line is the median. Flankers are 1.5 times the inter-quartile range from the nearest quartile. In addition, the mean and standard error of the mean (SEM) are plotted as solid dots with error bars.

In order to investigate whether there were differences in facially expressed emotions before, during, and after the activity, the mean automatically grade emotion scores were entered in a 8 (emotion: angry, disgusted, happy, interest, neutral, sad, scared, surprised) × 3 (event: before, during, after) ANOVA treating both factors as repeated measures. Summary statistics for this analysis are presented in Figure 3. This analysis revealed a significant main effect of emotion, *F*(3.94, 90.53) = 19.19, *p* < 0.001, $\eta_{p}^{2}$ = 0.45, while the main effect of event did not become significant, *F*(1.61, 37.07) = 1.28, *p* = 0.285, $\eta_{p}^{2}$ = 0.05. The emotion × event interaction, however, also showed a significant result, *F*(5.29, 121.61) = 3.87, *p* = 0.002, $\eta_{p}^{2}$ = 0.14. The Greenhouse-Geisser corrected analyses are reported because a test of sphericity showed that this assumption was violated.

The significant interaction shows that there were differences in how strongly emotions changed during movement as compared to before or after the activity between the emotions. We therefore followed up the analysis using *post hoc* tests. Our research question was focused on comparing online emotions (during the activity) to those expressed before or after the activity, and we therefore limited the analyses to comparing mean emotions before and during activity and during and after activity (i.e., we did not compare emotions before and after the activity directly). We set up the *post hoc* procedure in the following way: We calculated separate, one-way ANOVAs for each of the emotions and setup ANOVA contrasts such that before and after the downhill event were compared against facially expressed emotions during the activity. We report both unadjusted and Bonferroni-adjusted *p* (across the set of eight analyses). We report the unadjusted *p* in an exploratory spirit but urge the reader to treat these results cautiously.

The first hypothesis predicts that participants will express less happiness during the activity. There was some evidence in favor of this hypothesis as scores of happiness were reduced during downhill movement, *t*(46) = −2.09, *p*_unadjusted_ = 0.041, *p*_bonf_ = 0.33, even though this effect did not survive Bonferroni adjustment. Further, we found that facial display of anger was increased during downhill movement, *t*(46) = 2.87, *p*_unadjusted_ = 0.0061, *p*_bonf_ = 0.049, and this effect was significant also after adjusting for multiple comparisons. Finally, facial display of disgust may have been reduced during riding, *t*(46) = −1.96, *p*_unadjusted_ = 0.056, *p*_bonf_ = 0.45, even though this conclusion is only weakly supported by an unadjusted *p* that is marginally above threshold.

There were no further effects for any of the other facially expressed emotions when comparing before/after against during the activity (all *p*_unadjusted_ > 0.12). For a further description of the results, see Figure 3 and Table 1.

**Table 1:** Summary of a hierarchical linear regression model with interest as the dependent variable and all other facially expressed emotions and part during the activity as predictors.

Emotions	*b*	β	SE	*t*
Neutral	−0.06	−0.04	0.16	−0.4
Happy	−0.05	−0.03	0.14	−0.37
Sad	−0.06	−0.03	0.17	−0.33
Angry	0.37	0.25	0.18	2.06^*^
Surprised	0.58	0.44	0.11	5.51^***^
Scared	0.29	0.12	0.17	1.68
Disgusted	0.23	0.18	0.09	2.51^*^
part	−0.05	−0.13	0.03	−1.69
(Intercept)	0.26	–	0.14	1.89

Degrees of freedom (*DF*) = 159; *p < 0.05; ***p < 0.001; *b*, raw regression coefficient; β = standardized regression coefficient; SE, standard error.

### Facially Expressed Interest

**Figure 4:**
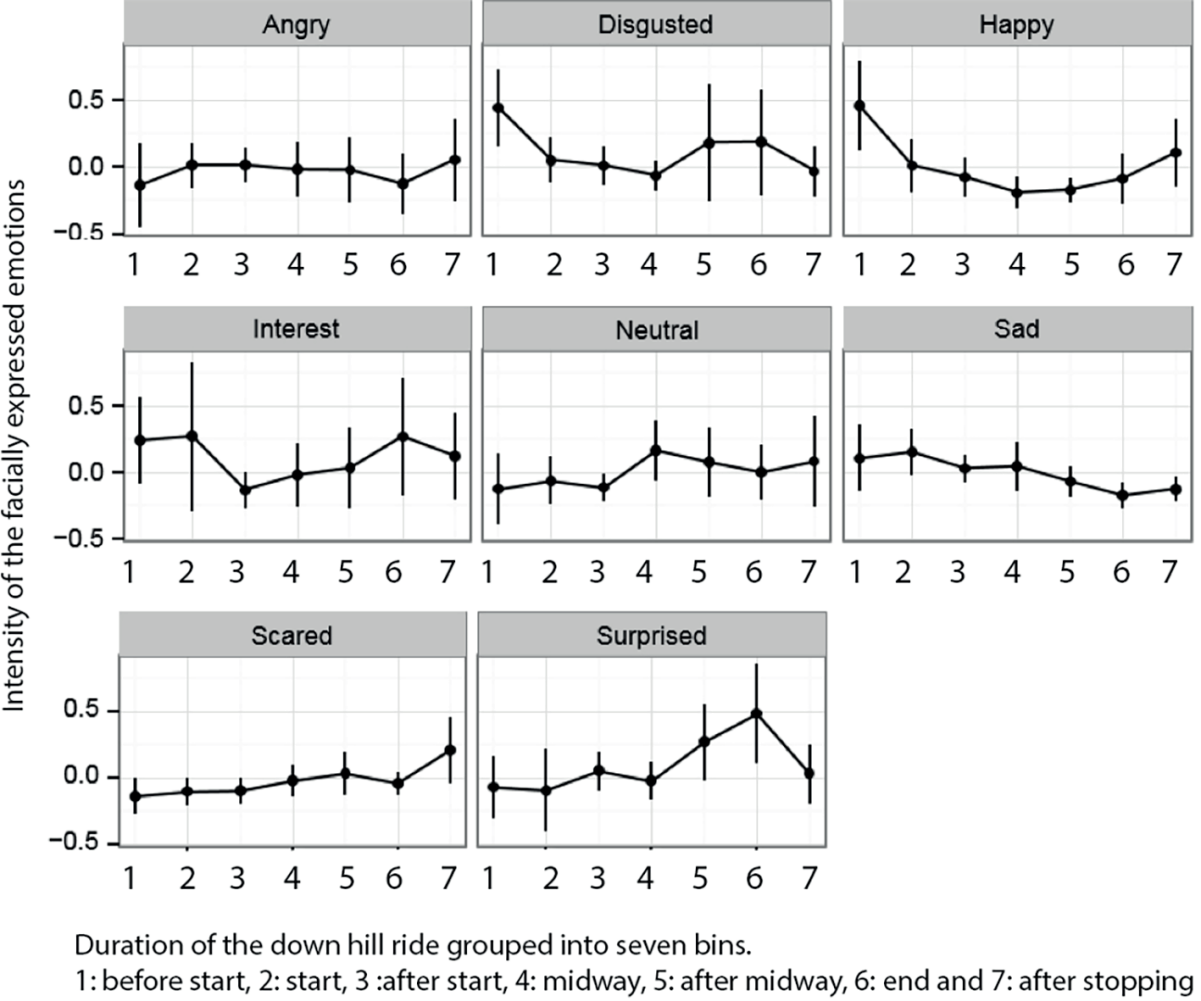
The downhill event was divided into seven subsequent episodes, and the line in each frame shows the intensity of its respective emotion throughout the event. The dots along the lines reflect the *z*-transformed mean scores for the facially expressed emotions during each of the seven episodes, and error bars are 95% confidence intervals (unadjusted).

The second hypothesis predicted higher levels of expressed interest during the activity than before and after. As seen in Table 1, and as reported in the previous paragraph, there were no significant differences in facially expressed interest between the events. To conduct a more in-depth analysis of the interest variable and its development throughout the downhill experience, we divided the stream of facially expressed emotion scores during the downhill activity into seven subparts per participant (segments classified as before and after the activity did not enter into this analysis). This approach was chosen because subjects differed in the total length of their rides. Figure 4 summarizes the development of the expressed emotions during the downhill ride. There is no clear pattern of the interest variable over the course of the ride. Some of the other emotions show interesting patterns. Happiness, for example, seems to be highest at the beginning and the end of the ride, possibly reflecting anticipatory and reflective effects. Also, both fear and surprise seem to increase during the run.

To investigate our interest variable more closely, we next conducted a hierarchical linear regression treating interest as the outcome and the other facially expressed emotions (as well as part during the downhill ride) as predictors. We added random intercepts for each subject and treated the predictors as fixed effects. This model revealed a positive relationship between interest and surprise, *b* = 0.58, *t*(159) = 5.51, *p* < 0.001; interest and anger, *b =* 0.37, *t*(159) = 2.05, *p* = 0.041; and interest and disgust, *b* = 2.23, *t*(159) = 2.5, *p* = 0.013. There was no relationship between the emotional expression of interest and the emotional expression of sadness as we predicted *a priori* based on the overlap in the involved AUs.

### Facial Movement Versus Self-Report

Our third hypothesis predicted that there would not be any strong associations between the online measure (facially expressed emotions) and the retrospective measure (self-reported state emotions). First, based on the BEST-questionnaire administered after the activity, we calculated mean scores for self-reported happiness (mean of self-reports on “happiness,” “satisfaction,” and “pleasure”) as well as interest (mean of self-reports on “interest,” “engagement,” and “enthusiasm”). We then normalized the self-reports to fall into the interval [0,1] to be comparable to the facially expressed emotion scores and entered them together with the main facially expressed emotions during the activity into a 2 (measure: FaceReader vs. BEST) × 5 (emotion: happy, angry, interest, sad, scared) ANOVA, treating both factors as repeated measures. The results of this analysis are graphically depicted in Figure 4.

**Figure 5:**
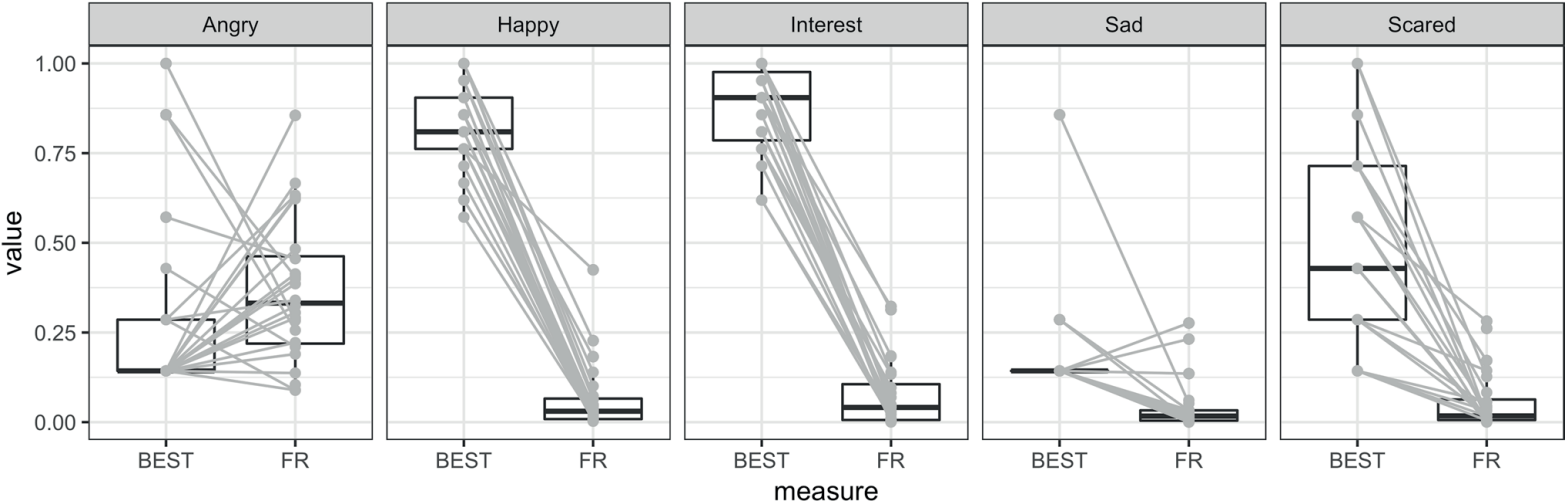
Comparing self-reported emotions after the ride (BEST) and facially expressed emotions (FR). There are obvious differences in both interest, happiness and fear, all of which are much higher when measured using self-reports when compared to facially expressed emotions. Gray lines are individual participants to illustrate repeated measures.

We observed significant effects of measure, *F*(1, 23) = 292.46, *p* < 0.001, $\eta_{p}^{2}$ = 0.93, and emotion, *F*(2.76, 63.55) = 38.14, *p* < 0.001, $\eta_{p}^{2}$ = 0.62, as well as a significant interaction between the two, *F*(2.47, 56.84) = 71.99, *p* < 0.001, $\eta_{p}^{2}$ = 0.76. The Greenhouse-Geisser corrected analyses are reported when appropriate. Given the significant interaction effect, we followed up this analysis using *post hoc* paired *t* tests between self-reported and facially expressed emotions (see Figure 5). Happiness was more frequently expressed in *post hoc* self-reports than facially during the activity, *t*(23) = 27.1, *p*_bonf_ < 0.001. Interest was also higher in self-reports than facial expressions, *t*(23) = 28.7, *p*_bonf_ < 0.001. Similarly, self-reported sadness was increased relative to expressed emotions, *t*(23) = 4.3, *p*_bonf_ < 0.001 as was self-reported fear, *t*(23) = 8.37, *p*_bonf_ < 0.001. Self-reported and facially expressed anger, however, were not different, *t*(23) = −1.03, *p*_bonf_ = 1.0.

Finally, we wanted to see whether it was possible to predict emotions that were reported after the activity by including only facially expressed emotions from during the activity. We conducted a set of multiple regression analysis where each of the nine self-reported emotion variables served as dependent variables and the facially expressed emotions as independent variables. In order to encourage exploratory results, no adjustment for multiple comparisons was applied and any significant results would therefore have a highly increased chance to be a false positive. However, none of the 8 × 9 = 72 regression coefficients were significantly different from zero, suggesting either that facially expressed emotions are unrelated to self-reported emotions, or that the momentary emotional feelings of a strenuous event are different from the overall feelings experienced when the event has been completed.

## Discussion

The present article investigated the momentary emotions during, and the retrospective emotions immediately after, an intense downhill mountain-bike run. As predicted, the results showed that the participants expressed less momentary happiness while riding the bike than before the ride and also a trend showing less happiness after they had stopped. This replicated the main finding to Hetland et al. (2018), which demonstrated that backcountry skiers display more happiness when they stop skiing than while they ski. Contrary to our hypothesis, facially expressed interest was less intense during the event than the levels before and after. We also found that the facially expressed emotions during the event were unrelated with the self-reported emotions given immediately after the event. Finally, we found that the participants expressed surprisingly high levels of anger.

Our first finding shows that the participant expresses less happiness during the event than before the event, and although not significant, the trend shows less happiness also after the event. This corresponds with the explanation provided by the flow theory, which argues that while in flow, the participants are too immersed in the activity to consciously register the enjoyment created by the feeling system (Csikszentmihalyi, 1999). More precisely, the execution of a difficult task keeps the attention strictly focused on performing each step of the behavioral sequences, reducing the minds’ capacity to attend to the accompanying stream of feelings. However, the feelings are still there (Damasio, 2018).

This finding is also compatible with the explanation provided by functional wellbeing approach (FWA; Vittersø, 2016). The FWA argues that people do have emotional experiences during the flow. However, these experiences are not characterized by enjoyment, but rather by feelings of interest, immersion, and engagement. Indeed, feeling interested is in itself a facilitator of a concentrated awareness. The happiness experienced in retrospect, however, does not arise as the sum of the feelings experienced during the activity, but rather as an evaluation of goal accomplishment for the activity as a whole. The first part of the FWA argument was not supported by the present results, but the data are consistent with the second part of the argument.

Within extreme sport, fear is an important emotion. It forces the participants to be alert to potential hazards, to stay focused, and to take necessary counter measures (Brymer and Schweitzer, 2013). However, Brown and Fraser (2009) argue that there is a misconception in the literature about fear as a motive for taking part in extreme sport. They argue that it is mastery and learning that is the main motivation. Willig’s (2008) perspective offers support to this finding as she argues that the main motivation is both developing skills and the experience of positive emotions. The FWA (Vittersø, 2016) argues that these two motives might be connected. If the challenges posed by an activity slightly outweigh the present level of skills, this will elicit interest and communicate to the mind that some mental and/or bodily efforts are required in order to for a particular goal to be achieved. Thus, in a challenging situation like downhill mountain biking during which the participants must push themselves to the very edge of their capabilities, the FWA predicts that interest rather than happiness is the most functional feeling state. The second hypothesis in this study therefore predicted that the participants would express more interest during the downhill ride than after the activity was over. However, in contrast to what was expected, the results showed that interest also decreased during the activity.

To further inspect the emotional change during the activity, the facial expressed data were divided into seven subsequent episodes. A close inspection of these episodes revealed that happiness decreased as the activity progressed. In the last part of the trip, however, happiness started to increase, although only to a para-significant (*p* = 0.07) level. One reason why the post-event happiness was not significantly different from the event happiness might have to do with the short period of post-event measures. The facially expressed emotions were only measured for about 10 s after the downhill trip stopped. The facially expressed interest did not return a clear pattern, and the analysis also revealed extensive variation in all seven sections.

In a previous study by Hetland and Vittersø (2012) exploring emotional experiences in BASE jumping and skydiving, the participants reported a total absence of self-reported sadness. In a later article investigating emotional experiences in backcountry skiing, utilizing the same technology as in the present article, facially expressed sadness was reported to be the second most prominent emotion after happiness (Hetland et al., 2018). Facially expressed sadness has striking similarities with facially expressed interest as described by Campos et al. (2012).

This lead Hetland et al. (2018) to speculate that facially expressed sadness might partly be mislabeled interest. This assumption was not confirmed in the present study. Facially expressed interest (operationalized according to Campos et al., 2012) did not show any correlation with facially expressed sadness. In addition, facially expressed sadness was the least prominent of the facially expressed emotions. These findings cast doubt to whether facially expressed interest can be measured with the action unit proposed by Campos and coworkers – at least in a sample of downhill bikers using the present technology.

The final hypothesis of the present study predicts that the momentary emotions during a strenuous event are governed by a different system than the emotions experienced after the event has stopped. Thus, no strong association between online emotions and retrospective emotions is to be expected. According to the FWA (Vittersø, 2018), momentary emotions are generated by a non-evaluative feedback process in which the level of difficulty inherent in the ongoing activity determines the quality of the accompanying feeling state. As for the emotions experienced after the activity has been completed, an evaluative process of comparing the result of the activity with the goals of the event determines the post-event emotion. A successful result will generate positive emotions, whereas an unsuccessful result will lead to negative emotions.

The findings from the current article show that there is no relation between the online emotions and self-reported post-event emotions. These data are thus in line with the final hypothesis. However, our results can only show that self-reports and facially expressed emotions diverge across subjects and do not prove that such relationship does not exist. It is indeed notoriously difficult to prove the null hypothesis and in order to do so, we would need to apply Bayesian statistics or frequentist equivalence testing. Unfortunately, our sample size is not large enough for such an approach. Our results must therefore be interpreted with appropriate caution.

The high levels of facially expressed anger observed in the current study were somewhat surprising. Anger was actually the most prominent of the facially expressed emotions before and during the activity. Furthermore, there was a significant reduction in anger after the activity was over. This finding was not predicted in our hypotheses. In retrospect, a possible explanation might be drawn from the association between anger and obstacles. The emotion of anger is typically elicited when an active plan is frustrated or an external source is obstructing the path toward goal achievement (Oatley, 1992). However, this explanation cannot properly account for the anger expressed before the cycling started, except, perhaps, in the form of an anticipation of possible hindrances ahead. Another reason for the result could be attributed to the fact that that facially expressed anger is characterized by lowered eyebrows and tightened eyelids, not unlike an expression of concentration. If facially expressed anger instead represents concentration, this would explain the high levels before and during, and a reduction after the activity was over. We readily admit that this explanation is somewhat speculative, and future investigations will hopefully throw more light on this surprising, but interesting, observation.

Before the cycling event, participants also displayed higher levels of disgust than they did later on. Again, this was unexpected and might be due to some kind of unconscious rejection or aversion toward the upcoming event. Rejections are, at least in general, the most common elicitor of disgust (Rozin et al., 2008). Once more, this is a suggestive interpretation, and further studies are certainly needed before conclusions can be drawn.

### Limitations and Further Research

This article applied a design with automated facial coding of emotions during the high arousal activity of a downhill mountain-bike run. Even though this method opens up new possibilities, it is not without shortcomings. First, to the technical challenge of capturing facial expressions, we found that the rapid shifts in light conditions made it challenging to analyze some of the films and consequently leading to issues with missing data.

Further, in order to be able to correctly capture facially expressed emotions, the participants were instructed not to use any kind of eye protection, which again could have affected their performance during the event. In addition, the camera had to be placed in the participants’ field of vision. Although most participants said they quickly habituated, these factors might have affected their display of facial expressions. As these challenges demonstrate, measuring emotions is by no means an easy task. Even though facial expression is regarded an reliable measure of momentary emotions, one needs to be cautious when translating facial movement into an emotion-laden component.

We also recorded relatively short-time segments in pre and post (a range of 15–30 s), which may cause a challenge when comparing the facially expressed emotions pre/post to during (2.5-min range).

Technically, film is nothing more than still images displayed in rapid sequence, and FaceReader also analyzes each frame as a still image. The validity of FaceReader’s ability to read and correctly analyze still images is well established. Still, we would welcome a validation study with video as input to confirm this.

Finally, knowledge is still meager when it comes to analyze emotions that are not “basic” in the Ekman sense of the term. Currently, FaceReader categorizes these as “neutral,” but this “grab-bag” category may have emotional content not captured by the FACS system. In spite of its shortcomings, however, this technology gives us a first peek into how an intense activity like mountain biking is experienced as it unfolds – an opportunity that has been unavailable until now.

## Conclusion

The current article contributes to the literature in several ways. By utilizing a newly developed method for capturing facial expressions that are associated with expressed emotions by use of small video cameras and existing technology for automatic facial coding, the article provides moment-to-moment measures of expressed emotions. Further, it tests a new way of measuring facial expressed interest. However, the data cast serious doubts about whether interest can be measured according to the current description – at least in the present setting and with the current technology.

We believe more confidence can be ascribed to the results reported about happiness, and that an important contribution from the current study is the reduced level of happiness our participants expressed as they biked downhill. The happiness typically reported from these kinds of high-aroused activity can most likely be attributed to the happy feelings observed before the biking event, and the high levels of happiness recorded after the biking had ended. So in the end, we may contribute to explaining why extreme sport athletes report happiness as one of their main motives. Not because extreme sport is an activity filled with momentary happiness, but because it creates an opportunity for development and growth – and that feels good and makes you happy.

## Ethics Statement

This study was carried out in accordance with the recommendations of Norwegian Center for Research Data.

## Author Contributions

AH is responsible for the study and writing. EK is responsible for all data collection and substantial part of the writing. MM contributed to all statistical analyses and written the result section. JV contributed significantly to the study design, theoretical development, and writing.

### Conflict of Interest Statement

The authors declare that the research was conducted in the absence of any commercial or financial relationships that could be construed as a potential conflict of interest.
